# PD-1/PD-L1抑制剂在晚期肿瘤患者中的相关肺炎发生率和发生风险：一项荟萃分析

**DOI:** 10.3779/j.issn.1009-3419.2020.103.14

**Published:** 2020-11-20

**Authors:** 康 陈, 步彤 孙

**Affiliations:** 130031 长春，吉林大学中日联谊医院肿瘤血液科 Department of Oncology and Hematology, China-Japan Union Hospital of Jilin University, Changchun 130031, China

**Keywords:** PD-1/PD-L1抑制剂, 免疫相关性肺炎, 荟萃分析, 免疫检查点抑制剂, PD-1/PD-L1 inhibitors, Immune-related pneumonia, *Meta*-analysis, Immune checkpoint inhibitor

## Abstract

**背景与目的:**

免疫检查点抑制剂（immune checkpoint inhibitor, ICI）对绝大多数晚期肿瘤具有良好的疗效，给晚期肿瘤患者带来了新的希望，然而由ICI激活的免疫系统，可能攻击人体正常组织器官导致相应的免疫毒性，如ICI相关肺炎。本研究对程序性细胞死亡受体1（programmed cell death protein 1, PD-1）抑制剂及程序性细胞死亡受体配体1（programmed cell death protein ligand 1, PD-L1）抑制剂在晚期肿瘤患者中的相关肺炎发生率和发生风险进行荟萃分析。

**方法:**

计算机检索PubMed、Cochrane Library、EMbase、中国知网数据库，收集PD-1/PD-L1抑制剂在晚期肿瘤患者中相关肺炎发生率的研究，检索时限为2000年1月-2020年1月。用Revman 5.3软件及R 3.6.2软件进行统计学分析比较不同情况下的肺炎发生率。

**结果:**

共纳入15篇研究，包含8, 642例患者。其中治疗组为PD-1或PD-L1抑制剂，对照组为化疗组，所有级别免疫性肺炎发生率的风险比（odds ratio, OR）为6.63，而高级别为4.87；ICI组非小细胞肺癌中所有级别肺炎发生率是其他瘤种的1.658倍，而高级别肺炎为2.299倍；二线及以上应用ICI所有级别肺炎的发生率是一线的0.489倍，且高级别肺炎的发生率是一线及以上应用ICI的0.449倍。

**结论:**

与化疗相比，PD-1和PD-L1抑制剂发生相关肺炎的风险较高，在ICI组中非小细胞肺癌以及一线应用中有较高发生风险。本研究为晚期肿瘤的临床治疗用药及并发症防治提供指导依据。

## Abstract (en)

**Background and objective:**

Immune checkpoint inhibitors (ICIs) have good efficacy on most advanced tumors, which brings new hope to patients with advanced tumors. However, the immune system activated by ICIs may attack human normal tissues and organs, resulting in corresponding immunotoxicity, such as checkpoint inhibitor pneumonitis. This article carried out a *meta*-analysis on the incidence and risk of programmed cell death protein 1 (PD-1) and programmed cell death protein ligand 1 (PD-L1) inhibitor-associated pneumonia in advanced tumors patients.

**Methods:**

The computer retrieval of PubMed, Cochrane Library, EMbase and CNKI was performed, and the studies on the occurrence rate of PD-1/PD-L1 inhibitor-associated pneumonia in terminal cancer patients were collected, with the retrieval time limit of January 2000 to January 2020. Statistical analysis was conducted by using Revman 5.3 and R 3.6.2 software to compare the occurrence rate of pneumonia under different circumstances.

**Results:**

15 studies were included, involving 8, 642 patients, of which those with PD-1/PD-L1 inhibitor were treatment group, and those with chemotherapy were control group. The odds radio of all grades of immune pneumonia was 6.63, and that of high grade was 4.87. The occurrence rate of all grades of pneumonia in the ICI group with non-small cell lung cancer (NSCLC) was 1.658 times than other tumors, and that of high grade was 2.299 times. The occurrence rate of all grades of pneumonia in second-line or more treatment with ICI was 0.489 times than that in first-line, and that of high grade was 0.449 times than that in first-line or more treatment with ICI.

**Conclusion:**

Compared with chemotherapy, the risk of immune-associated pneumonia is higher in PD-1 and PD-L1 inhibitors, and its occurrence risk is high in the ICI group with NSCLC and the first-line treatment with ICI. This paper provides guidance for clinic treatment of terminal cancers and prevention of complications.

过去十年间，肿瘤的免疫治疗飞速发展，其中免疫检查点抑制剂（immune checkpoint inhibitor, ICI）已成为肿瘤治疗领域的热点，并且在疗效方面取得令人瞩目的成绩。ICIs主要靶点包括细胞毒性T淋巴细胞抗原4（cytotoxic T lymphocyte-associated antigen 4, CTLA-4）和程序性细胞死亡受体1（programmed cell death protein 1, PD-1）及程序性细胞死亡受体配体1（programmed cell death protein ligand 1, PD-L1）。ICIs通过抑制CTLA-4、PD-1及PD-L1，解除免疫抑制，活化T淋巴细胞，达到清除肿瘤的目的^[1]^，其中PD-1抑制剂（Nivolumab、Pembrolizumab）和PD-L1抑制剂（Atezolizumab），已被作为多种类型晚期肿瘤的推荐用药写入指南。然而，随着ICIs在临床全面的应用，由此产生的免疫相关不良事件（immune related adverse events, irAE）也逐渐得到关注。这些irAEs主要包括皮肤、肺脏、内分泌、血液、消化系统等，其中免疫检查点抑制剂肺炎（checkpoint inhibitor pneumonitis, CIP）是ICI相关毒性的一种。CIP的总体发生率虽然不高，但是严重的CIP造成免疫治疗中断，甚至成为威胁生命的重要因素之一。因此临床医生需要对这一罕见但严重的不良事件给予更多的关注^[2]^。

即使已有众多临床试验关于PD-1/PD-L1抑制剂相关肺炎发病率的报道，但上述临床试验中所获得的个体及队列数量较为局限。此外，虽然部分相关荟萃分析也研究了免疫相关肺炎的发生率，然而也面临着纳入分析的研究数量及人数有限的问题。基于此，我们对PD-1/PD-L1抑制剂在晚期癌症患者中相关肺炎的临床试验进行了荟萃分析，并比较了免疫相关性肺炎在不同肿瘤类型及治疗方案间的危险度和发病率，旨在为后期的临床治疗及相关并发症的预防提供指导依据。

## 1 资料与方法

### 1.1 检索策略

根据系统综述和荟萃分析优先报告的条目（Preferred Reporting Items for Systematic Reviews and *meta*-Analyses, PRISMA）原则，用计算机检索PubMed、Cochrane Library、EMbase、中国知网数据库，检索针对PD-1/PD-L1抑制剂在晚期肿瘤患者中相关肺炎的发生率及危险度的研究的相关文献，因avelumab和durvalumab发表报告较少，故检索词英文为：“immune checkpoint inhibitor”、“ICIs”、“nivolumab”、“pembrolizumab”、“atezolizumab”、“cancer”、“tumor”、“phase Ⅱ”、“phase Ⅲ”、“pneumonia”、“pneumonitis”，中文为：“PD-L1抑制剂”、“PD-1抑制剂”、“免疫检查点抑制剂”、“特瑞普利单抗”、“卡瑞利珠单抗”，搜索时间从2000年1月-2020年1月，文献类型限制为临床试验，为防止文献漏检，同时搜索相关参考文献，本文未进行手动检索。

### 1.2 纳入标准

对符合下列标准的文献，将其纳入该荟萃分析：①研究对象为公开发表的晚期恶性肿瘤的Ⅱ期和Ⅲ期随机对照试验；②研究对象接受PD-1或PD-L1抑制剂，对照组为常规化疗组；③可以获得所有级别（1级-4级）和高级别（3级-4级）肺炎的相关数据；④纳入的研究中研究对象总数不少于200例；⑤研究为中、英文文献。

### 1.3 排除标准

① 回顾性研究、病例报道、Ⅰ期临床试验；②重复发表、综述、文摘、讲座、荟萃分析；③纳入研究中的PD-1抑制剂或PD-L1抑制剂组为联合治疗，PD-1抑制剂和PD-L1抑制剂同时出现；④文献信息不全或有误，无法提取有效信息。

### 1.4 资料提取

由两位研究人员独立进行文献质量评价、文献筛选、核对数据，进行文献的纳入及排除，然后整理文献的数据信息，对出现意见不同且该文献是否能够纳入的问题，需通过查阅相关资料及讨论或者邀请第三位研究人员一起解决问题。数据提取是在系统回顾和荟萃分析优先报告条目的基础上进行的。为每篇文章提取的数据包括：第一作者的姓名、发表年份、试验阶段、采用的盲法、可供分析的患者数量、治疗类型、肿瘤类型，以及两组人群中所有级别（1级-4级）和高级别（3级-4级）肺炎的例数。

### 1.5 纳入文献的质量评价

所纳入的随机对照试验按照Cochrane干预措施系统评价手册对纳入的文献进行整理并且进行质量评价。主要包括正确的随机方法、是否使用盲法、患者的选择、随机分配方案的隐藏、缺失数据报告、选择性报告研究结果和其他偏倚来源7个方面，由两位评价员进行评价，相关分歧通过协商解决。

### 1.6 统计学方法

本研究的主要目的是了解不同肿瘤类型PD-1/PD-L1治疗患者的肺炎发生率，并比较PD-1/PD-L1抑制剂与标准治疗方案的肺炎风险比（odds ratio, OR）以及95%置信区间。该研究需要对从文献中提取整理后的数据采用统计学软件Revman 5.3进行处理。为了更加直观比较不同条件下肺炎发生率，我们根据数据进行了肺炎发生率的单个率的处理，肺炎发生率的荟萃分析采用R 3.6.2软件中荟萃程序包的荟萃prop函数进行合并。发生率若不服从正态分布，进行对数转换。采用单因素*Logistic*回归模型对不同组间的肺炎发生率进行检验。用*I*^2^来评价纳入文章的异质性，经异质性检验，如果各研究间*I*^2^ < 50%，则采取固定效应模型计算其合并量；如果各研究间的*I*^2^≥50%，则选用随机效应模型进行合并量的计算，并需要对其异质性来源进行亚组分析或敏感性分析等，从而分析出其异质性的来源；若考虑纳入文献样本量不多，无法进一步进行亚组分析，需通过分析其特征及查阅资料找出引起各项研究间异质性高的其他来源。采用*Begg*和*Egger*法生成漏斗图评价相关发表偏倚。显著性水平α设置为0.05，*P* < 0.05时定义为差异有统计学意义。

## 2 结果

### 2.1 文献筛选

从数据库检索到431篇潜在的文章，其中29篇由于重复发表而被排除后剩余402篇，对剩余文献中综述、文摘、讲座、荟萃分析一并去除。剩余文章进行了标题和摘要的筛选，根据我们的入选标准，347篇文章被剔除，55篇文章进行了全文筛选，40篇文章由于数据不全、评价指标不同、样本量少的原因被删除。最后，我们最终纳入了15篇^[3-17]^文章，共8, 642例患者，其中PD-1/PD-L1抑制剂组为4, 785例，对照组为3, 857例。详细的文献纳入及排除见流程图（图 1）。

**图 1:**
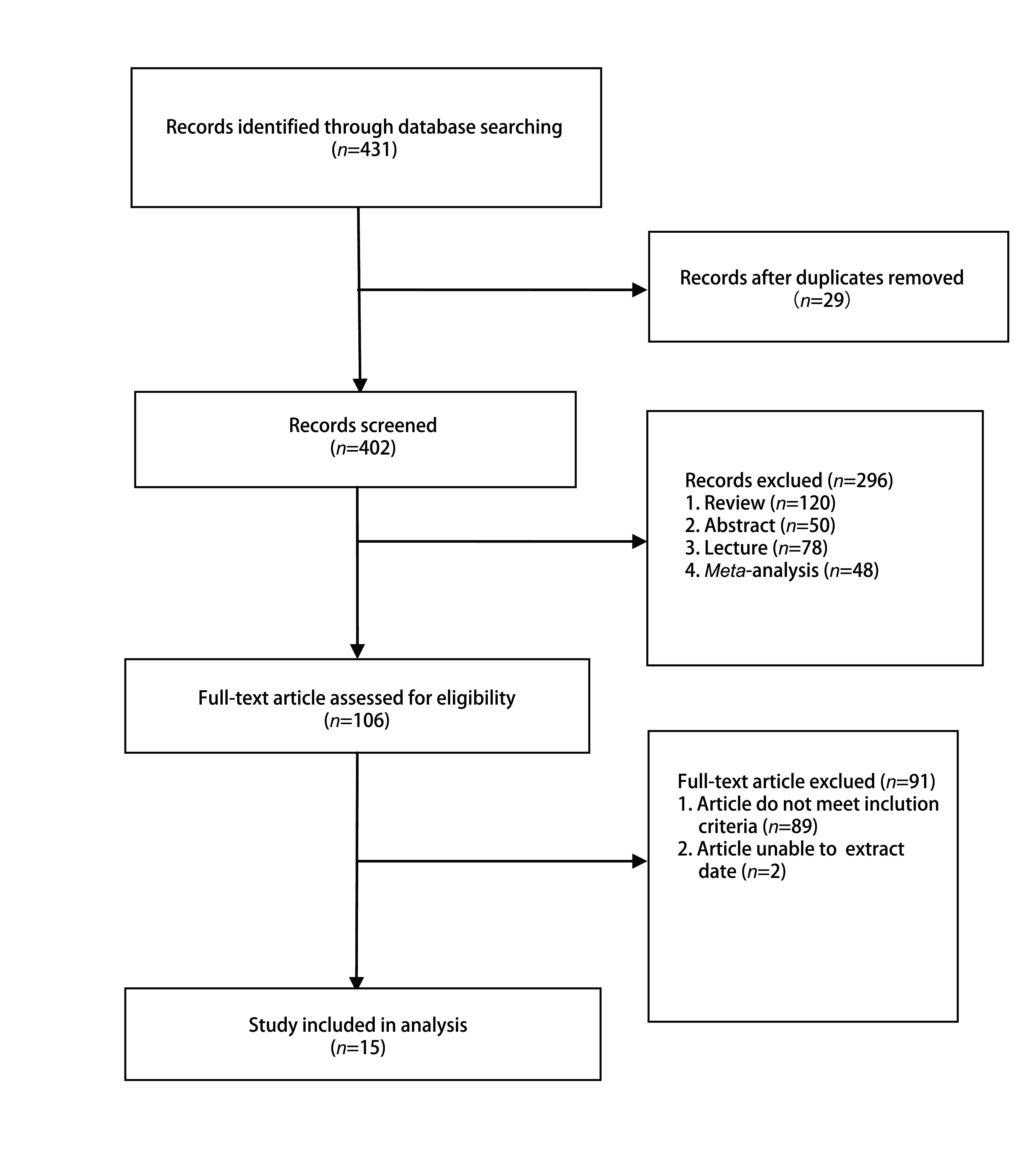
筛选文献纳入的流程图 Flow diagram of the study selection process

### 2.2 纳入文献特征

本研究共纳入15篇文献，全为英文文献，共纳入患者8, 642例，其中PD-1/PD-L1抑制剂组为4, 785例，对照组为3, 857例，其中采用Nivolumab的6篇，采用Pembrolizumab的7篇，采用Atezolizumab的2篇。对文献资料进行提取，整理研究的基本情况及提取数据（表 1）。

**表 1:** 纳入研究的基本特征 Characteristics of the included studies

Included studies	Author/Year	Trials design											Patients characteristics		
		Type of study	Phase of study	Experimental arm					Control arm				Type of cancer	Disease stage	Line of therapy
				Drug	Dosage	Patients (n)	Age (yr)		Drug	Patients (n)	Age (yr)				
Checkmate 017	Brahmer *et al*./2015^[3]^	RCT	3	Nivolumab	3 mg/kg q14	131	62		Docetaxel	129	64		NSCLC	Ⅲb or Ⅳ	2^nd^ line
Checkmate 057	Borghaei *et al*./2015^[4]^	RCT	3	Nivolumab	3 mg/kg q14	287	61		Docetaxel	268	64		NSCLC	Ⅲb or Ⅳ	2^nd^ line
Checkmate 066	Robert *et al*./2015^[5]^	RCT	3	Nivolumab +Placebo	3 mg/kg q14 placebo q21	206	64		Dacarbazine +Placebo	205	66		Melanoma	Ⅲ or Ⅳ	1^st^ line
Checkmate 037	Weber *et al*./2015^[6]^	RCT	3	Nivolumab	3 mg/kg q14	268	59		Chemotherapy	102	62		Melanoma	ⅢC or Ⅳ	2^nd^ line
Checkmate 141	Ferris *et al*./2016^[7]^	RCT	3	Nivolumab	3 mg/kg q14	236	59		Cetuximab or Docetaxel or Methotrexate	111	61		Scchn	Recurrent or Ⅳ	2^nd^ line
Checkmate 026	Carbone *et al*./2017^[8]^	RCT	3	Nivolumab	3 mg/kg q14	271	63		Chemotherapy	270	65		NSCLC	Ⅲb or Ⅳ	1^st^ line
Keynote 024	Reck *et al*./2015^[9]^	RCT	3	Pembrolizumab	200 mg q21	154	64.5		Chemotherapy	150	66		NSCLC	Ⅳ	1^st^ line
Keynote 002	Ribas *et al*./2015^[10]^	RCT	2	Pembrolizumab	2 mg/kg q21	178	62		Chemotherapy	171	63		Melanoma	Ⅲ or Ⅳ	≥2^nd^ line
					10 mg/kg q21	179	60						
Keynote 010	Herbst *et al*./2016^[11]^	RCT	3	Pembrolizumab	2 mg/kg q21	339	63		Docetaxel	309	62		NSCLC	Advanced	2^nd^ line
					10 mg/kg q21	343	63						
Keynote 045	Bellmunt *et al*./2017^[12]^	RCT	3	Pembrolizumab	200 mg q21	270	67		Chemotherapy	272	65		Urothelial Carcinoma	Advanced	2^nd^ line
Keynote 042	Tony *et al*./2019^[13]^	RCT	3	Pembrolizumab	200 mg q21	637	63		Chemotherapy	627	63		NSCLC	Advanced	1^st^ line
Keynote 061	Kohei *et al*./2019^[14]^	RCT	3	Pembrolizumab	200 mg q21	296	62.5		Paclitaxel	296	60		Gastric carcinoma	Advanced	2^nd^ line
Keynote 040	Ezra *et al*./2019^[15]^	RCT	3	Pembrolizumab	200 mg q21	247	60		Cetuximab or Docetaxel or Methotrexate	248	60		Scchn	Recurrent or Ⅳ	2^nd^ line
Oak	Rittmeyer *et al*./2016^[16]^	RCT	3	Atezolizumab	1, 200 mg q21	425	63		Docetaxel	425	64		NSCLC	Ⅲb or Ⅳ	2^nd^ line
Polar	Fehrenbache *et al*./2016^[17]^	RCT	3	Atezolizumab	1, 200 mg q21	144	63		Docetaxel	143	62		NSCLC	Ⅲb or Ⅳ	2^nd^ line
NSCLC: non small cell lung cancer; RCT: randomized controlled trial; Scchn: squamous cell carcinoma of head and neck.															

### 2.3 纳入文献的质量

根据随机对照试验研究Cochrane干预措施系统评价手册，对纳入的文献进行评价。根据偏倚风险评估图可得知我们纳入的15项研究总体偏倚风险被评估为低风险，所有研究的质量都是合格的（图 2A和图 2B）。

**图 2:**
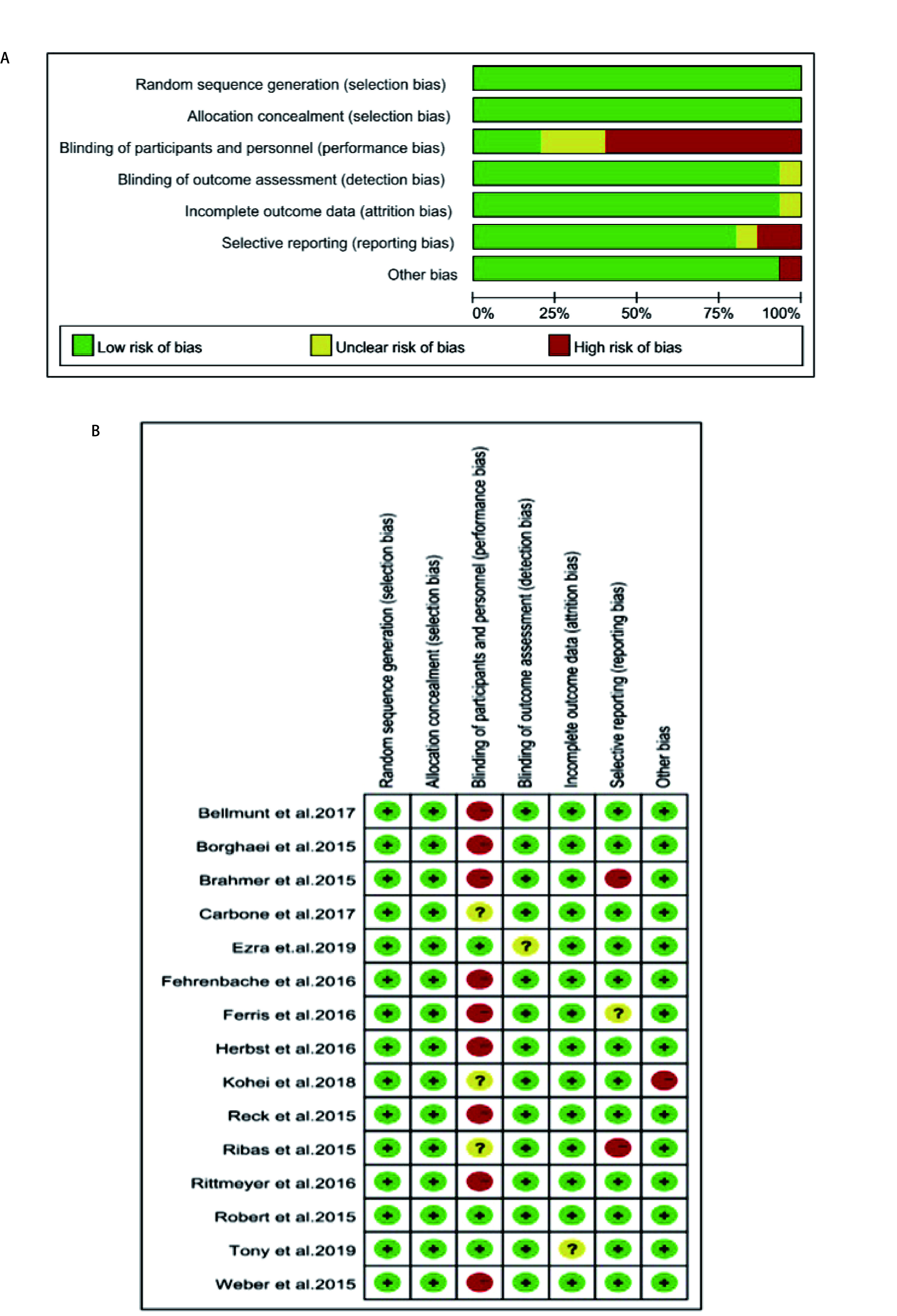
在所有纳入的研究中，不同类别的偏倚风险图和总结。A：偏倚风险图；B：偏见风险总结。 Risk of bias graphs and summaries in various categories across all of the studies included. A: risk of bias graph; B: risk of bias summary.

### 2.4 荟萃分析结果

#### 2.4.1 CIP的发生率

研究的实验组为PD-1抑制剂或PD-L1抑制剂，通过筛选文献及统计数据获得所有级别（1级-4级）肺炎发生率为1.0%-8.0%，高级别（3级-4级）肺炎发生率为0.6%-3.0%（表 2）。进一步应用R 3.6.2软件中荟萃程序包的荟萃prop函数进行合并，得出所有级别免疫性肺炎总的发生率为3.0%（95%CI: 2.0%-4.0%）（图 3）。

**表 2:** 纳入研究的肺炎发生率 Incidence of pneumonia in the studies

Study	Evaluable patients			Pulmonary toxicity											
				Any-grade						High-grade				
	Anti-PD-1/PD-L1	Control		Anti-PD-1/PD-L1			Control			Anti-PD-1/PD-L1			Control	
				Events(*n*)	Incidence		Events(*n*)	Incidence		Events(*n*)	Incidence		Events(*n*)	Incidence
Checkmate 017^[3]^	131	129		6	4.60%		1	0.80%		1	0.80%		1	0.80%
Checkmate 057^[4]^	287	268		8	2.80%		1	0.30%		3	1%		0	0
Checkmate 066^[5]^	206	205		3	1.50%		0	0		0	0		0	0
Checkmate 037^[6]^	268	102		6	2.20%		0	0		0	0		0	0
Checkmate 026^[7]^	267	263		7	2.60%		0	0		4	1.5%		0	0
Checkmate 141^[8]^	236	111		5	2.11%		1	0.90%		2	0.80%		0	0
Keynote 024^[9]^	154	150		9	5.80%		1	0.70%		4	2.60%		1	0.70%
Keynote 002^[10]^	357	171		6	1.70%		0	0		2	0.60%		0	0
Keynote 010^[11]^	682	309		31	4.50%		6	1.90%		14	2.10%		2	0.60%
Keynote 045^[12]^	266	255		11	4.10%		1	0.40%		6	2.30%		0	0
Keynote 042^[13]^	636	615		53	8.00%		3	0.50%		22	3%		1	0.20%
Keynote 061^[14]^	294	276		8	3.00%		0	0		2	0.70%		0	0
Keynote 040^[15]^	246	234		10	4.00%		3	1%		3	1%		3	1%
Oak^[16]^	609	578		6	1.00%		1	0.20%		4	0.70%		0	0
Poplar^[17]^	142	135		4	2.80%		0	0		1	0.70%		0	0
Total	4, 781	3, 801		173	3.60%		18	0.50%		68	1.40%		8	0.20%
PD-1: programmed cell death protein 1; PD-L1: programmed cell death protein ligand 1.														

**图 3:**
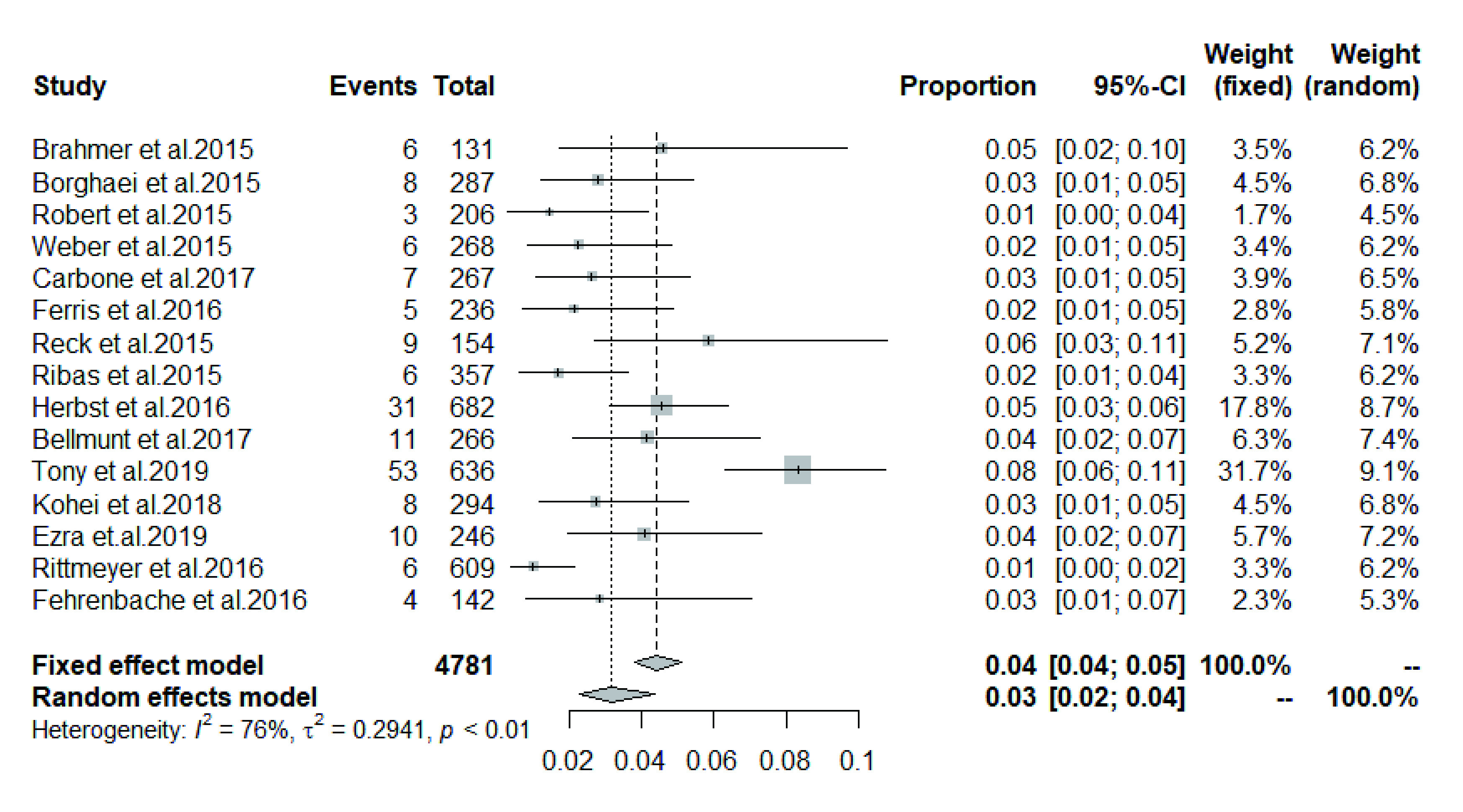
PD-1/PD-L1治疗组所有级别肺炎发生率 Incidence of pneumonia at all levels in PD-1/PD-L1 treatment group

#### 2.4.2 所有级别和高级别肺炎发生率的OR值

对实验组为PD-1抑制剂或PD-L1抑制剂，对照组为化疗组的研究的肺炎发生率进行评估，所有级别肺炎发生率OR值为6.63（95%CI: 4.22-10.40, *P* < 0.000, 01），此分析无异质性（*I*^2^=0%）（图 4A）。高级别肺炎发生率OR值为4.87（95%CI: 2.64-8.99, *P* < 0.000, 01），此分析同样无异质性（*I*^2^=0%）（图 4B）。

**图 4:**
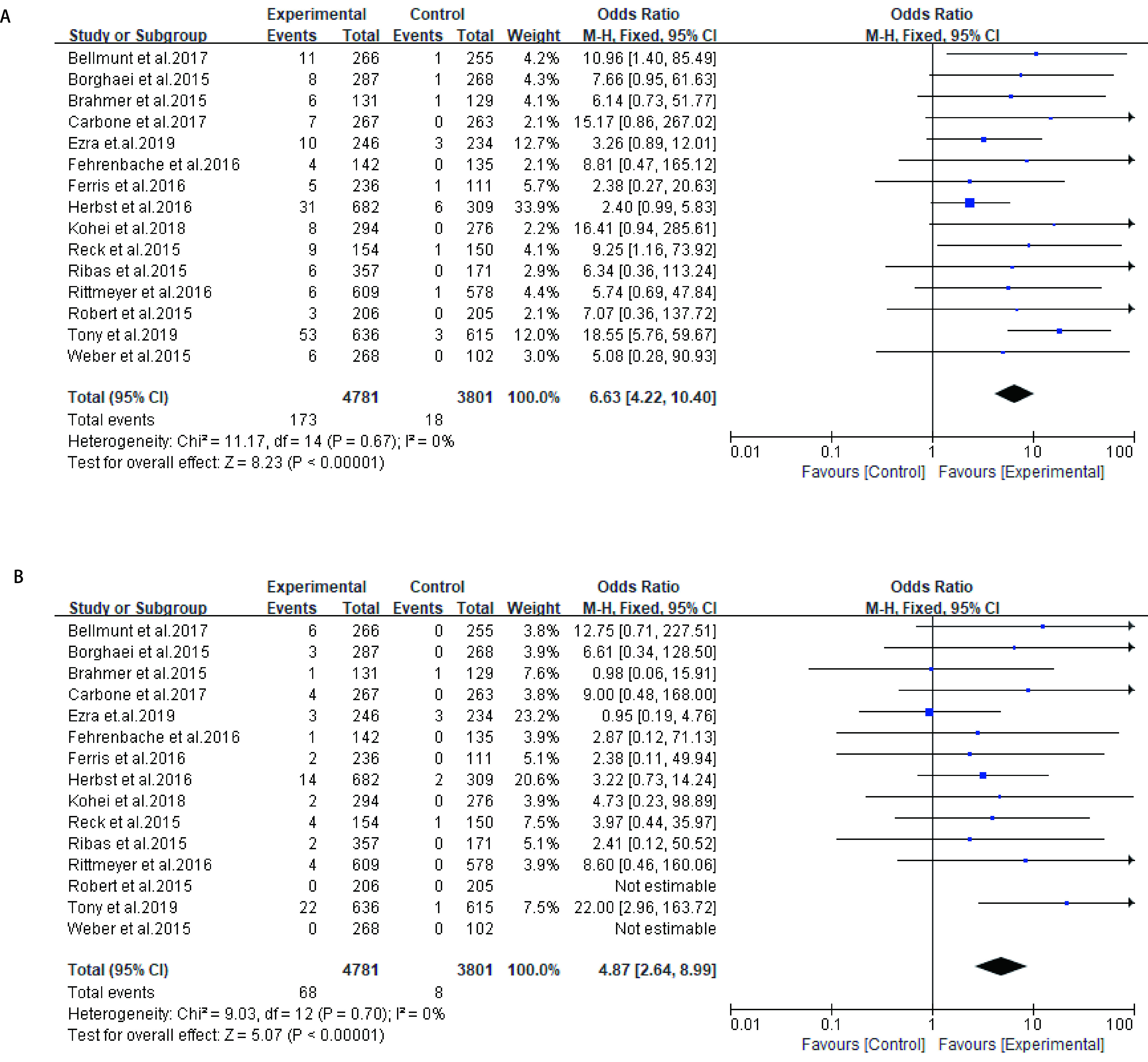
PD-1/PD-L1抑制剂治疗组与对照组肺炎OR值。A：所有级别肺炎；B：高级别肺炎。 OR value of pneumonia in PD-1/PD-L1 inhibitor treatment group and control group. A: all-grade of pneumonia; B: high-grade pneumonia. OR:odds ratio.

亚组分析：同时评估了不同类型免疫检查点抑制剂（Nivolumab、Pembrolizumab、Atezolizumab）相关肺炎的风险（图 5A和图 5B）。与对照组相比，Nivolumab的应用增加了所有级别肺炎的风险（OR=6.27, 95%CI: 2.35-16.73, *P*=0.000, 2），但高级别肺炎的风险未显示增加（OR=3.89, 95%CI: 0.99-15.34, *P*=0.05）。Pembrolizumab治疗组增加所有级别肺炎的风险（OR=6.72, 95%CI: 3.95-11.43, *P* < 0.000, 01），同时高级别肺炎的风险增加（OR=5.06, 95%CI: 2.45-10.42, *P* < 0.000, 1）。Atezolizumab治疗组所有级别肺炎的风险增加（OR=6.75, 95%CI: 1.22-37.45, *P*=0.03），但高级别肺炎的风险未增加（OR：5.74, 95%CI: 0.69-47.86, *P*=0.11）。最后Nivolumab、Pembrolizumab和Atezolizumab在所有级别组（*P*=0.99）和高级别组（*P*=0.93）均未发现明显差异。

**图 5:**
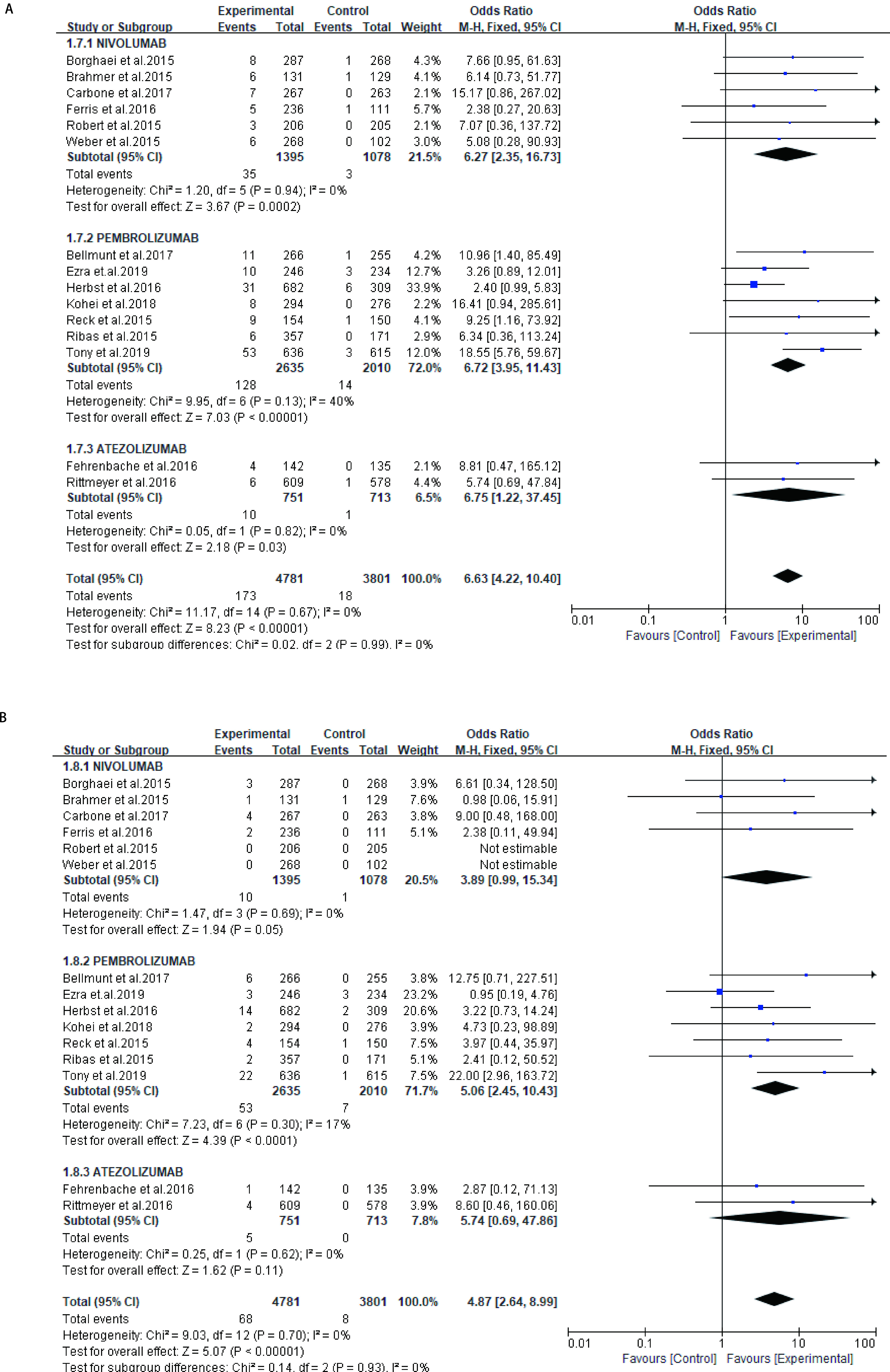
不同类型ICIs相关肺炎的OR值。A：所有级别肺炎；B：高级别肺炎。 OR values for pneumonia associated with different types of ICIs. A: all-grade of pneumonia; B: high-grade pneumonia. ICIs: immune checkpoint inhibitors.

#### 2.4.3 ICIs实验组非小细胞肺癌和其他瘤种的CIP发生率的比较

荟萃非小细胞肺癌中所有级别CIP的发生率为4.0%（95%CI: 2.0%-6.0%），其中*I*^2^=82%，对该荟萃分析进行敏感性分析后发现当剔除Keynote042时*I*^2^=0%，逐一剔除各个研究重新行荟萃分析后的结果在4.0%-5.0%之间，与未剔除前的荟萃分析比较，差异不大，提示此荟萃分析较稳健，此分析包含研究数量较少，无法进一步行亚组分析，故采用随机效应模型（图 6A），其他瘤种中所有级别CIP的发生率：3.0%（95%CI: 2.0%-4.0%）（图 6B）。将发生率代入*Logistic*回归模型检验后得出ICIs治疗组非小细胞肺癌中所有级别CIP的发生率是其他瘤种的1.658倍（95%CI: 1.193-2.340, *P*=0.003, 2），结果有统计学意义。非小细胞肺癌中高级别CIP的发生率：2.0%（95%CI: 1.0%-3.0%），其*I*^2^=55%，敏感性分析后剔除Keynote042时*I*^2^=6.2%，逐一剔除各个研究重新行荟萃分析后的结果均在2.0%左右，与未剔除前的荟萃分析比较，差别不大，提示此荟萃分析较稳健。Keynote042的治疗组所有级别CIP发生率为8%，Keynote042的研究对象中纳入了一部分肺部有轻度慢性疾病的患者，同时，纳入的对象均为一线应用免疫检查点抑制剂的患者，据报道免疫性肺炎的发生率一线应用ICI高于二线应用ICI^[18]^，不同的患者纳入标准可能导致了观察到的异质性，最终选择随机效应模型（图 6C），其他瘤种高级别肺炎发生率为1.0%（95%CI: 1.0%-2.0%）（图 6D）。代入*Logistic*回归模型检验得出治疗组非小细胞肺癌中高级别CIP的发生率是其他瘤种的2.299倍（95%CI: 1.327-4.236, *P*=0.004, 6），差异有统计学意义。

**图 6:**
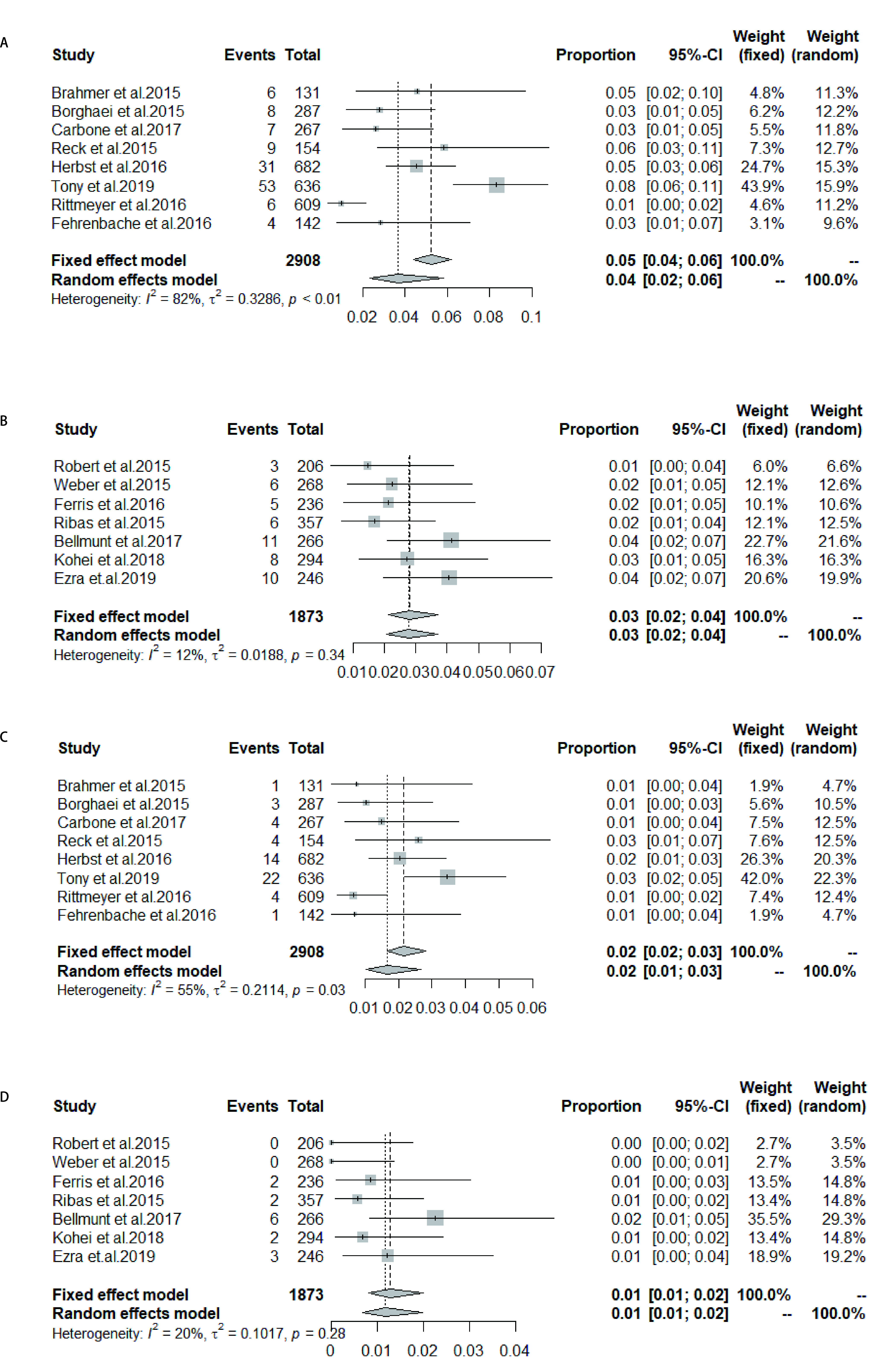
ICIs组非小细胞肺癌中肺炎发生率（A：所有级别肺炎；C：高级别肺炎）；ICIs组其他瘤种中肺炎发生率（B：所有级别肺炎；D：高级别肺炎） Incidence of pneumonia in non-small cell lung cancer in the ICIs group (A: all-grade of pneumonia; C: high-grade pneumonia); Incidence of pneumonia in other tumors of ICIs group (B: all-grade of pneumonia; D: high-grade pneumonia)

#### 2.4.4 一线和二线及以上应用ICIs免疫相关性肺炎的发生率比较

一线应用ICIs中所有级别CIP的发生率为4.0%（95%CI: 2.0%-9.0%），*I*^2^=82%，进行敏感性分析后，逐一剔除各个研究重新行荟萃分析后的结果在3.0%-5.0%之间，与未剔除前的荟萃分析比较差别不大，提示此荟萃分析较稳健。逐一剔除研究后*I*^2^均未低于50%，但研究数量较少，无法进一步亚组分析，考虑异质性来源有：①研究的免疫检查点药物不同；②研究的瘤种不同；③研究应用为同一药物，但应用的药物剂量不同，最终采用随机效应模型（图 7A）。二线及以上应用ICIs中所有级别CIP的发生率为3.0%（95%CI: 3.0%-4.0%）（图 7B）。将发生率代入回归模型检验后得出二线及以上应用ICIs中所有级别CIP的发生率是一线应用ICIs的0.489倍（95%CI: 0.359-0.668, *P* < 0.000, 1），故差异有统计学意义。一线应用ICIs中高级别CIP的发生率为3.0%（95%CI: 2.0%-4.0%）（图 7C）。二线及以上应用ICIs中高级别CIP的发生率为1.0%（95%CI: 1.0%-2.0%）（图 7D）。代入*Logistic*回归模型检验得出二线及以上应用ICIs中高级别CIP的发生率是一线应用ICIs的0.449倍（95%CI: 0.277-0.732, *P*=0.001, 2），结果差异有统计学意义。

**图 7:**
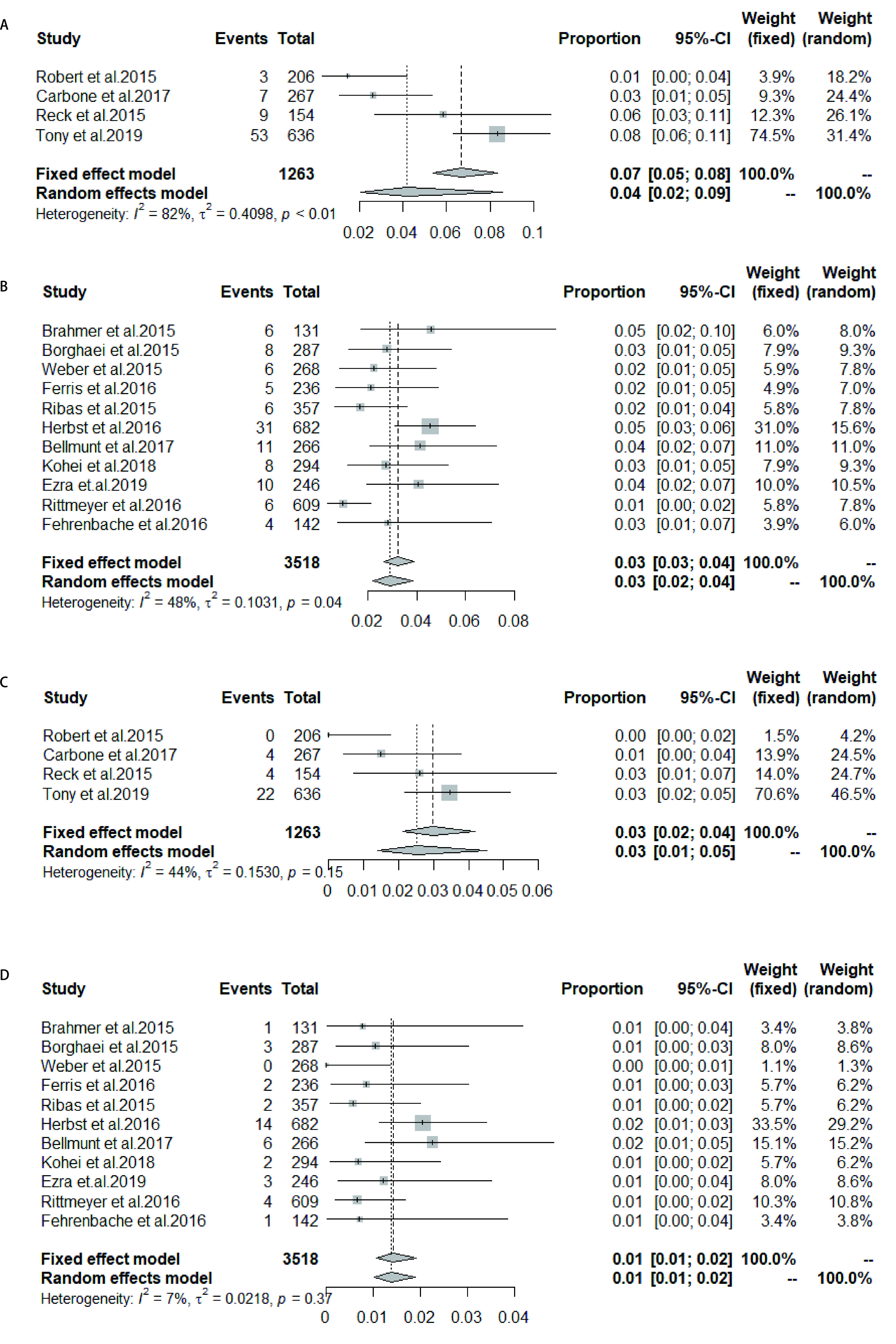
一线应用ICIs中肺炎的发生率（A：所有级别肺炎；C：高级别肺炎）；二线及以上应用ICIs中肺炎的发生率（B：所有级别肺炎；D：高级别肺炎） Incidence of pneumonia in front-line ICIs (A: all-grade of pneumonia; C: high-grade pneumonia); Incidence of pneumonia in second-line and above (B: all-grade of pneumonia; D: high-grade pneumonia)

### 2.5 发表偏倚

纳入的15项研究在所有级别肺炎和高级别肺炎的OR值的荟萃分析中均表现为对称，无发表偏倚（图 8）。

**图 8:**
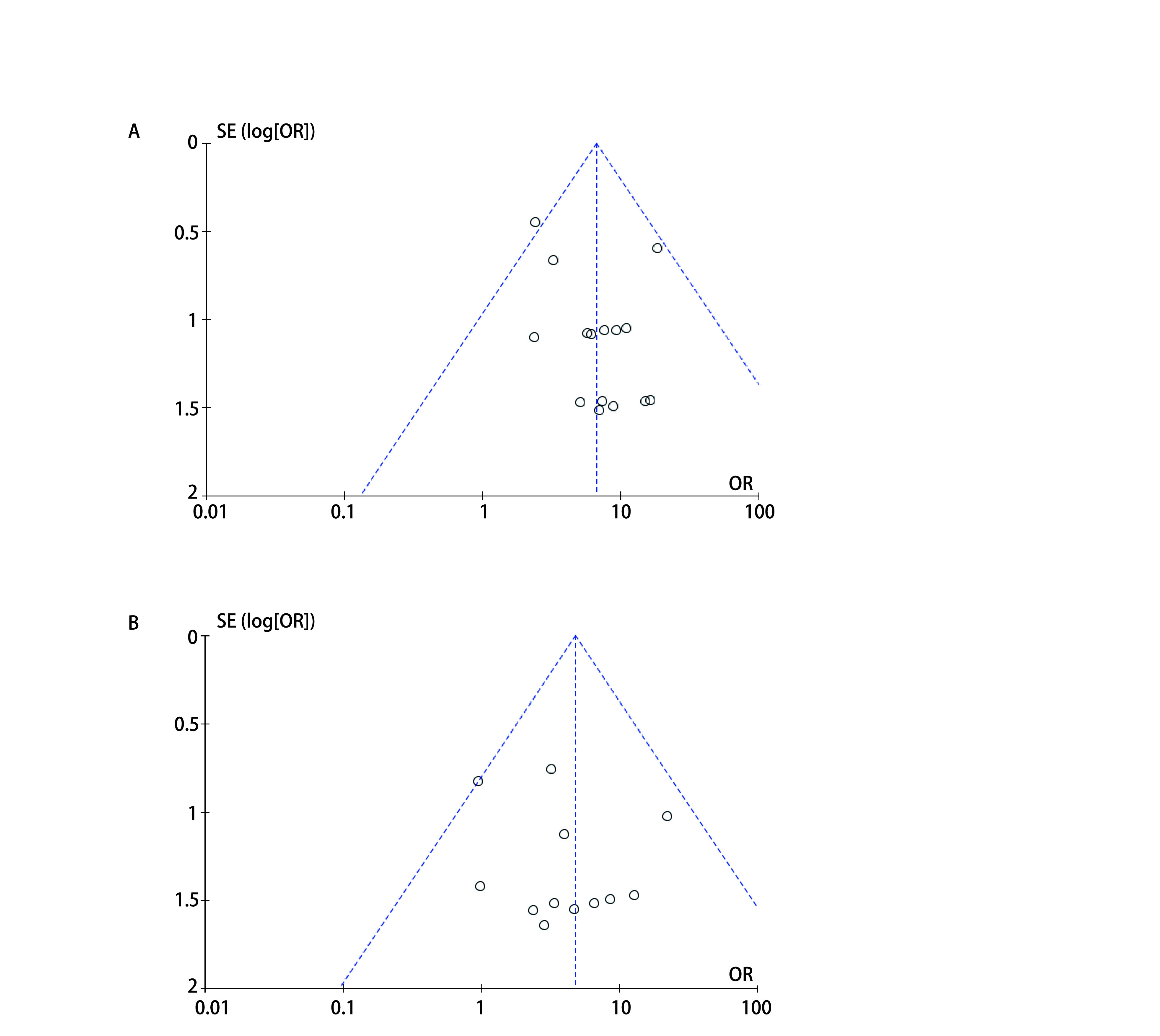
相关肺炎的OR值的漏斗图。A：所有级别肺炎；B：高级别肺炎。 The funnel plot of the OR of pneumonia. A: all-grade of pneumonia; B: high-grade pneumonia.

## 3 讨论

近年来，ICIs已成为最受欢迎的各种类型晚期肿瘤治疗方案之一，但免疫相关的不良反应是完全不同于传统的放疗、化疗及靶向治疗的不良反应。它们具有独特的特征，包括特定器官的倾向性、没有剂量依赖的相关性以及潜在的迟发性^[19]^。在这些与免疫相关的不良反应中，CIP往往是严重的，并可能危及生命。因此，对CIP的深入了解，对临床治疗及并发症防治有重要意义。

CIP主要的临床症状是咳嗽、呼吸困难及呼吸急促，发生CIP的中位时间为2.5个月（范围：0.8个月-11.0个月）。在所有CIP病例中，72%的患者为1级-2级。与甲状腺炎和肝炎等自限性免疫反应不同，大部分的CIP需要激素或免疫抑制剂的治疗，据文献^[20]^报道，超过85%的患者可以通过停药和免疫抑制治疗得到缓解或治愈，但也有一部分患者使用激素治疗之后得不到缓解，一项*meta*分析^[21]^显示，PD-1/PD-L1抑制剂致死的主要原因为CIP（35%）。有关报道^[22]^指出：不同瘤种的CIP的发生率存在差异，相比恶性黑色素瘤患者，使用ICIs的非小细胞肺癌及肾恶性肿瘤患者CIP的发生率明显更高，分别为4.1% *vs* 1.6%（*P*=0.002）和4.1% *vs* 1.6%（*P* < 0.001），本荟萃分析同样发现，非小细胞肺癌患者中CIP的发生率较高。不同瘤种的CIP发生率差异的具体机制尚不明确，可能的原因：肿瘤微环境、免疫浸润、适应性免疫反应和新抗原的形成可能受到组织学的影响^[23]^。同时，ICIs治疗前接受过胸部放疗，接受EGFR-TKI联合ICIs治疗的驱动基因敏感突变阳性的非小细胞肺癌患者^[24, 25]^，先前存在慢性阻塞性肺疾病（chronic obstructive pulmonary disease, COPD）、肺纤维化等或目前存在肺部活动性感染的患者为发生CIP的高危人群^[20, 26]^，应用ICIs时需提高警惕。CIP的临床症状多种多样，所以一旦接受PD-1和PD-L1抑制剂的患者出现呼吸系统症状，均应行胸部电子计算机断层扫描（Computed Tomography, CT）检查。影像学特征包括提示急性间质性肺炎、隐源性组织肺炎、超敏性肺炎或非特异性间质性肺炎^[27]^。如果CT结果不确定，应行支气管镜检查并行支气管肺泡灌洗，若发生CIP，灌洗液中可见大量淋巴细胞。

与化疗方案相比，免疫检查点抑制剂可显著增加任何级别和高级别的肺炎的发生率和风险。Nivolumab、Pembrolizumab、Atezolizumab、Avelumab、Durvalumab等虽然具有显著的疗效和良好的安全性并且已被美国食品药品监督管理局（Food and Drug Administration, FDA）批准用于治疗不同类型的癌症，然而，使用这些免疫检查点抑制剂治疗的患者发生CIP的风险是一致的，临床医生在选择此类药物时应始终考虑到这一点^[28-31]^。

本研究分析的局限性首先表现为其他ICIs和其他肿瘤类型的覆盖不充分。因为在本研究收集数据时缺乏相关研究的数据，此文章的分析集中在PD-1/PD-L1抑制剂的相关肺毒性，而不包括其他ICI，如CTLA-4抑制剂。其次，文章的研究集中于黑色素瘤、非小细胞肺癌、胃癌、头颈部鳞癌和尿路上皮癌，对于其他类型的肿瘤，如结直肠癌或淋巴瘤，由于发表的报告数量有限、样本量小，所以没有被包括在内。另外，我们荟萃分析的研究中选择的患者是一组经过筛选的活动状态良好的患者，但是在临床实践中，器官功能障碍患者的实际毒性发生率可能更高。最后，荟萃分析的研究中有一部分患者先前存在肺间质疾病，这都可能造成研究的异质性，通过亚组分析和敏感性分析找到异质性来源，可证明荟萃分析的稳健性。值得注意的是，相关报道^[32]^指出CIP发生率与PD-1抑制剂的剂量无关，所以我们将同一研究的中不同剂量的PD-1抑制剂相关肺炎发生人数进行了合并，并不影响荟萃分析最终结果。针对PD-1/PD-L1通路的不同药物的肺炎发病率，还需要更多数据以及进一步的研究。根据此荟萃分析，我们建议在临床采用PD-1/PD-L1抑制剂治疗晚期肿瘤患者时，应注意患者的个体化管理，根据不良反应发生率的差异性，做好预防和相应的治疗准备。
